# *SLC22A4* Gene in Hereditary Non-syndromic Hearing Loss: Recurrence and Incomplete Penetrance of the p.C113Y Mutation in Northwest Africa

**DOI:** 10.3389/fgene.2021.606630

**Published:** 2021-02-10

**Authors:** Chiara Chiereghin, Michela Robusto, Lucia Mauri, Paola Primignani, Pierangela Castorina, Umberto Ambrosetti, Stefano Duga, Rosanna Asselta, Giulia Soldà

**Affiliations:** ^1^Humanitas Clinical and Research Center–IRCCS, Rozzano, Italy; ^2^Experimental Therapeutics Program, IFOM-FIRC Institute of Molecular Oncology Foundation, Milan, Italy; ^3^S. S. Genetica Medica, ASST Grande Ospedale Metropolitano Niguarda, Milan, Italy; ^4^Dipartimento di Scienze Cliniche e di Comunità, Università degli Studi di Milano and Fondazione IRCCS Cà Granda Ospedale Maggiore Policlinico, UO Audiologia, Milan, Italy; ^5^Department of Biomedical Sciences, Humanitas University, Pieve Emanuele, Italy

**Keywords:** non-syndromic sensorineural hearing loss, exome sequencing, *SLC22A4*, Northwest Africa, mutation, linkage analysis

## Abstract

Inherited hearing loss is extremely heterogeneous both clinically and genetically. In addition, the spectrum of deafness-causing genetic variants differs greatly among geographical areas and ethnicities. The identification of the causal mutation in affected families allows early diagnosis, clinical follow-up, and genetic counseling. A large consanguineous family of Moroccan origin affected by autosomal recessive sensorineural hearing loss (ARSNHL) was subjected to genome-wide linkage analysis and exome sequencing. Exome-wide variant analysis and prioritization identified the *SLC22A4* p.C113Y missense variant (rs768484124) as the most likely cause of ARSNHL in the family, falling within the unique significant (LOD score>3) linkage region on chromosome 5. Indeed, the same variant was previously reported in two Tunisian ARSNHL pedigrees. The variant is present in the homozygous state in all six affected individuals, but also in one normal-hearing sibling, suggesting incomplete penetrance. The mutation is absent in about 1,000 individuals from the Greater Middle East Variome study cohort, including individuals from the North African population, as well as in an additional seven deaf patients from the same geographical area, recruited and screened for mutations in the *SLC22A4* gene. This study represents the first independent replication of the involvement of *SLC22A4* in ARSNHL, highlighting the importance of the gene, and of the p.C113Y mutation, at least in the Northwest African population.

## Introduction

Sensorineural hearing loss, resulting from malfunctions of the inner ear structures, is one of the most common congenital disorders in humans, affecting at least 1 in 1,000 newborns. It is estimated that about 60–70% of cases are due to genetic factors (Raviv et al., 2010); of these, about 75% are classified as non-syndromic hearing loss. Overall, inherited deafness is characterized by a high genetic heterogeneity, with more than 130 associated loci, about 100 different causal genes identified so far—most of which segregating with autosomal recessive sensorineural hearing loss (ARSNHL)—and many still to be discovered^1^. ARSNHL incidence is increased in countries with a high rate of consanguinity, such as the Great Middle East (GME) countries. Indeed, about 20–50% of all GME marriages are consanguineous, which is an approximately 100-fold higher rate compared with Western Europe and America (Scott et al., 2016). This has greatly contributed to the discovery of the genetic causes of inherited conditions, including hearing loss, although the genetic spectrum of ARSNHL-causing mutations in these countries remains poorly defined (Najmabadi and Kahrizi, 2014).

*SLC22A4* (OMIM^∗^604190) codes for the organic cation/carnitine transporter 1 (OCTN1), a 551-amino acid long protein belonging to the solute carrier family 22. It is composed of 12 α-helical transmembrane domains, a large glycosylated extracellular loop, as well as a big intracellular portion with predicted phosphorylation sites (Ben Said et al., 2016). OCTN1 is widely expressed in several tissues, including brain, small intestine, liver, kidney, and immune cells (Ishimoto et al., 2018). It functions as a plasma membrane transporter of different substrates, such as acetylcholine, carnitine (an important component in the transit of fatty acids to the mitochondrial inner membrane), and ergothioneine (ERGO), a potent naturally occurring antioxidant (Yabuuchi et al., 1999; Gründemann et al., 2005). In addition, there is also evidence of a specific OCTN1 localization in mitochondria (Lamhonwah and Tein, 2006).

To date, polymorphisms in the *SLC22A4* gene have been linked to Crohn’s disease (Peltekova et al., 2004; Kato et al., 2010; Xuan et al., 2012), colorectal cancer (Li et al., 2017), and susceptibility to rheumatoid arthritis (Tokuhiro et al., 2003; Lee et al., 2015), although the identified predisposing alleles only confer a modest increased risk. A founder homozygous missense variant (NM_003059.2:c.338G>A:p.C113Y, rs768484124) within *SLC22A4* was previously reported as the pathogenic mutation responsible for ARSNHL in two unrelated Tunisian families (Ben Said et al., 2016). In particular, an initial genome-wide linkage analysis in a large consanguineous family mapped the NSHL-causing gene within a 12.2 Mb critical region on chromosome 5 (5q23.2-31.1) corresponding to the *DFNB60* locus, with a maximum two-point LOD score of 4.13 for the most informative microsatellite marker (D5S658). Subsequently, exome sequencing of one affected individual was performed and a homozygous missense variant (c.338G>A; p.C113Y, rs768484124) in the *SLC22A4* gene, located within the critical region, was identified. The variant segregated with ARSNHL in the analyzed family and was detected, in the homozygous state and within the same haplotype, in a second deaf patient from an unrelated family (Ben Said et al., 2016). The mutation was demonstrated to affect a highly conserved cysteine residue within the first extracellular loop, which is predicted to contribute to protein stability and function, and to participate in the correct localization of the transporter to the apical plasma membrane (Ben Said et al., 2016).

Here, we report a detailed clinical and genetic study of a large consanguineous deaf family, of Moroccan origin, carrying the *SLC22A4* p.C113Y mutation, confirming its association with ARSNHL and its recurrence not only in Tunisia, but also in the Northwest African region.

## Materials and Methods

### Patients

**FIGURE 1:**
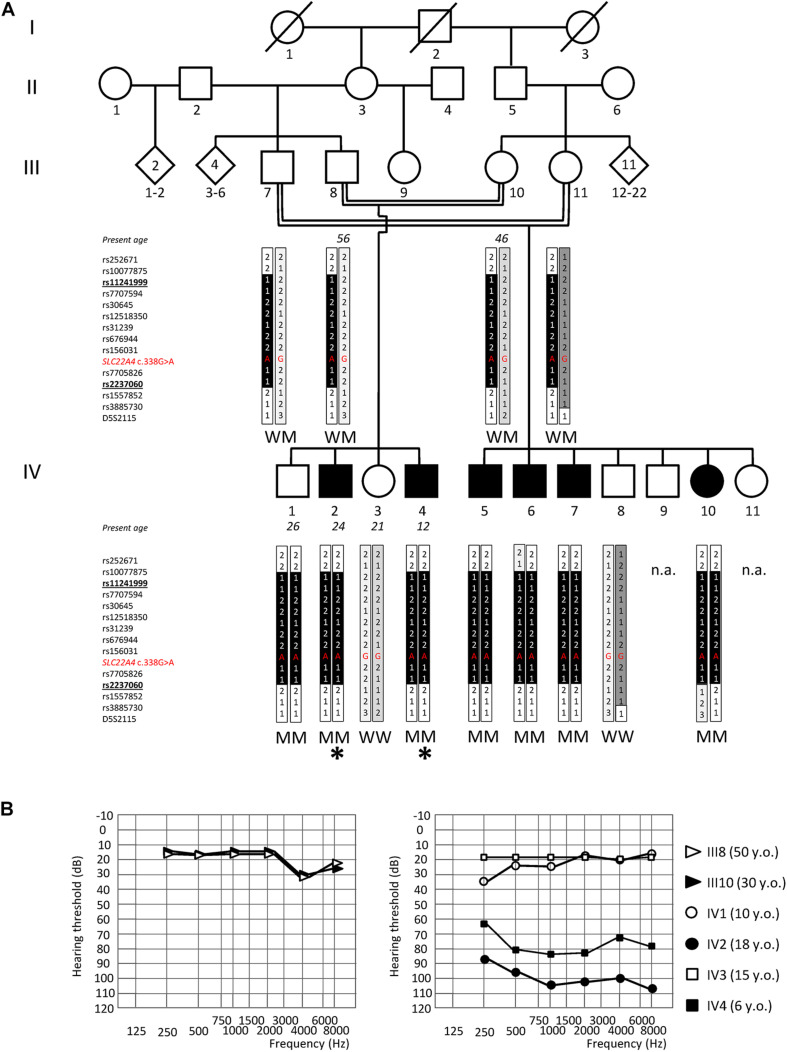
Genome-wide linkage analysis and audiograms of the NSHL7 family. **(A)** Pedigree showing the co-segregation of ARSNHL with a haplotype spanning from polymorphic marker rs11241999 to rs2237060 (bolded, underlined) within the chromosomal region 5q23.3–q31.1. The black vertical tract of the bar indicates the disease-associated haplotype shared by all affected individuals. The location within the haplotype and the corresponding genotype of the rs768484124 variant within *SLC22A4* (NM_003059.2:c.338G>A) is indicated in red. The genotype of available individuals is indicated below the corresponding symbols. Deaf individuals are represented by filled symbols and normal-hearing subjects by open symbols. The rhombus indicates an individual of unspecified sex. Individuals analyzed by exome sequencing are pointed by an asterisk. M, mutant (NM_003059.2:c.338G>A:p.C113Y); W, wild type. n.a.: not analyzed by genetic linkage study. **(B)** Audiograms of the normal-hearing parents (III8, III10; on the left) and of the four siblings (IV1–4) (average hearing loss for the right and left ears is shown). IV1 was not compliant for further audiometric evaluations. Age at audiometric evaluation is shown. y.o., years old.

The study on human subjects complies with the principles stated in the Declaration of Helsinki and was reviewed and approved by the Ethical Committees of the Niguarda Ca’ Granda Hospital and Fondazione IRCCS Cà Granda Ospedale Maggiore Policlinico. DNA samples were collected after having obtained a written informed consent from all participants and from parents of subjects younger than 18 years. Samples were anonymized, and sensitive data were managed according to the Italian legislation and the European General Data Protection Regulation (GDPR). One Moroccan consanguineous ARSNHL family (NSHL7), with multiple affected individuals (Figure 1A), was recruited. Seven additional unrelated ARSNHL individuals of North-African origin were selected from the patient cohort available at the Niguarda Ca’ Granda Hospital. For screening purposes, a cohort of 125 Italian subjects with no familiarity for hearing loss and instrumentally verified normal auditory function (mean age at withdrawal 32 ± 9 years) were included in the study.

Clinical history ruled out environmental factors as cause of deafness, and physical examination did not reveal any evidence of dysmorphic features. The family was routinely screened for mutations within gap-junction proteins connexins 26 and 30 (*GJB2*, *GJB6*) by Sanger sequencing and no pathogenic variant was detected. No family history of other diseases associated with variations in *SLC22A4* (Crohn’s disease, colorectal cancer, or rheumatoid arthritis) was reported. All patients underwent ear, nose and throat, as well as audiological examinations. Hearing levels were determined by pure-tone audiometry, in agreement with International Standard Organization (ISO 8253-1-3) protocols. Average thresholds in the range of 21–40 dB were defined as mild, 41–70 dB as moderate, 71–95 dB as severe, and >95 dB as profound hearing loss.

### Linkage Analysis

Genomic DNA was isolated from peripheral blood using a semi-automatic DNA extractor (Fujifilm Europe GmBH, Düsseldorf, Germany) according to standard protocols. Genotyping was performed on 13 individuals (III7, III8, III10, III11, IV1 to IV8, and IV10) from the NSHL7 family (Figure 1A), using the Infinium OmniExpressExome-8 v1.4 BeadChip array (Illumina, San Diego, CA, United States), which contains 958,497 markers, and following the manufacturer’s instruction. Image acquisition was performed with the iScan System (Illumina) and intensity files were converted into genotype calls with the Genome Studio software (Illumina). The raw genotyping data were filtered to remove (1) markers that did not give a signal; (2) uninformative (monomorphic) markers; (3) inconsistent markers; (4) multi-allelic SNPs; and (5) SNPs that were not annotated in dbSNPs. Finally, the genotyped markers were intersected with those present in the reference map selected for the analysis with GeneHunter2 (Illumina 300K HumanHap v2 deCODE, 318,230 markers): 184,190 SNPs were left to perform linkage analysis. Parametric multi-point data analysis was performed with the GeneHunter v2.1 r5 software (incorporated in the EasyLinkage Plus package)^2^ (Lindner and Hoffmann, 2005), using the following parameters: Allele frequencies, Codominant; Inheritance, Recessive; Chromosomes, All except X chromosome; distance between markers, 1 cM; HAP algo/SCFct, Viterbi/All; Frequency allele, 0.001; Used sib pair combination, all pairs of affected/phenotyped sibs; Scoring function, all; Eliminating less informative subject, OFF; Dumb IBD, OFF; Recombination counting, ON. Once the linkage peak on chromosome 5 was identified, a second analysis focused only on chromosome 5 was performed, resulting in a peak with LOD score = 3.716 (Supplementary Figure S1).

The microsatellite marker D5S2115 was also genotyped, using the assay available in the ABI PRISM_Linkage Mapping Set 2.5 (Applied Biosystems, Foster City, CA, United States). Amplification was performed by adding 5% DMSO to the PCR reaction, using the GoTaq DNA polymerase (Promega, Madison, WI, United States) and standard cycling conditions. The obtained fluorescently labeled alleles were analyzed on an automated ABI-3500 DNA sequencer (Applied Biosystems) to determine their size (allele 1 = 166 bp, allele 2 = 168 bp, allele 3 = 170 bp, Figure 1A).

### Exome Sequencing

Exome sequencing was performed on two affected siblings (IV2 and IV4; Figure 1A) starting from 1 μg of genomic DNA and using the SeqCap EZ Human Exome Library v.2.0 kit (Roche NimbleGen, Basel, Switzerland), following the manufacturer’s instructions. Paired-end 75 bp libraries were sequenced on a HiSeq 2000 (Illumina) at the service facility of the Yale Center for Genome Analysis. Quality control, alignment to the human reference genome (hg19, GRCh37 build), and variant calling were performed using the Whole-Exome sequencing Pipeline web tool (D’Antonio et al., 2013). Variant annotation was performed using ANNOVAR/wANNOVAR (Wang et al., 2010; Chang and Wang, 2012). Two different strategies for variant prioritization were used. For the analysis of the variants located within the identified linkage region on chromosome 5, the prioritization was performed after having selected common/shared variants. On the contrary, for the comprehensive analysis of exome data, the variant prioritization was conducted independently on each of the two siblings and candidate variants were first inspected separately before focusing on the shared ones. Potentially pathogenic variants were selected according to the following criteria: (1) variants with minor allele frequency (MAF) ≤ 1% in the 1,000 Genomes Project/GnomAD exome databases (1000 Genomes Project Consortium et al., 2015; Karczewski et al., 2020); (2) non-synonymous, splicing (i.e., all variants located within six nucleotides upstream or downstream of the exon/intron boundary), and insertion/deletion variants; (3) variants with a Combined Annotation Dependent Depletion (CADD) score ≥ 20 (Rentzsch et al., 2019); and (4) variants compatible with an autosomal recessive mode of inheritance (homozygous variations or ≥ 2 heterozygous variants in the same gene). When looking at variants located within the critical linkage region on chromosome 5, only homozygous ones were prioritized.

The rs768484124 variant within *SLC22A4* (NM_003059.2:c.338G>A; NP_003050.2:p.C113Y) was submitted to ClinVar (accession number SCV001450919).

### Sanger Sequencing

All exons and exon–intron boundaries of *SLC22A4* (GenBank accession number NM_003059), *P4HA2* (NM_001142599) exon 13, *SNX2* (NM_003100) exon10, *IK* (NM_006083) exon12, *PCDHB3* (NM_018937) exon1, and *PCDHB16* (NM_020957) exon1 were PCR amplified using sets of primers designed on the basis of their known genomic sequence. PCRs were performed on 10–20 ng of genomic DNA, following standard procedures. Primer sequences and thermal conditions for PCR amplification are available on request. Direct sequencing of amplified fragments was performed on both strands by means of the BigDye Terminator Cycle Sequencing Ready Reaction Kit v.1.1 and an automated ABI-3500 DNA sequencer (Applied Biosystem, Foster City, CA, United States). The Variant Reporter software (Applied Biosystems) was used for variant detection.

### High-Resolution Melting (HRM)

Screening for the *SLC22A4* c.338G>A (rs768484124) was performed on 125 audiologically tested normal-hearing controls. A specific primer pair was designed to PCR amplify a 265 bp genomic DNA fragment, encompassing the mutation site (primerF: 5′-CTGGCGCAACAACAGTGTC-3′; primerR: 5′-ACGCAGAGGGAGGGTCAG-3′). PCR amplicons were screened for mutations by HRM on a LightCycler 480 using the HRM Master kit (Roche, Basel, Switzerland) and a touch-down protocol. Thermal conditions for amplification are available on request. Amplicons were analyzed with the Gene Scanning Software (Roche).

## Results

### Linkage Analysis Points to a 3.2 Mb Peak on Chromosome 5

A genome-wide linkage study (comprising 958,497 markers) was performed on 13 individuals (III7, III8, III10, III11, IV1 to IV8, and IV10) from a large Moroccan consanguineous family (NSHL7) affected by post-lingual bilateral profound SNHL, with a likely recessive pattern of inheritance, in order to identify the genomic locus most likely to be involved in the disease pathogenesis. The probands are 24 (IV2)- and 12 (IV4)-year-old men with a normal-hearing brother (IV1) and sister (IV3) and four deaf (three males, IV5–7, and one female, IV10) and three unaffected (two males, IV8 and IV9, and one female, IV11) cousins (Figure 1A). Both probands had adequate speech development and are characterized by a moderate to profound bilateral hearing impairment affecting all frequencies (Figure 1B), which is treated with the use of hearing aids.

**FIGURE 2:**
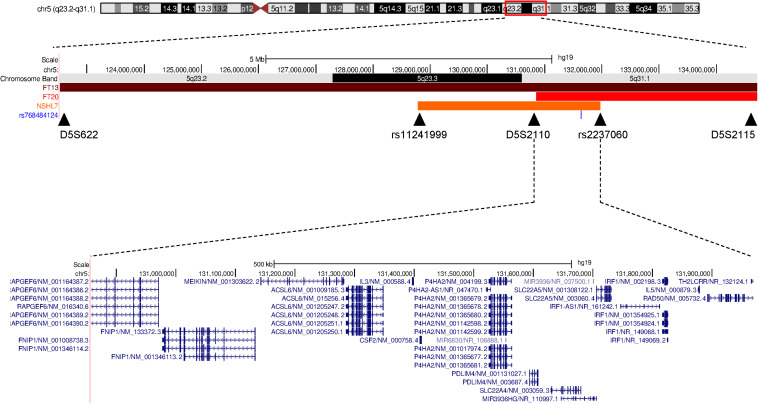
Schematic representation of the deafness-associated linkage region in the three North African families. A comparison between the linkage regions associated with the hearing impairment in the two previously reported Tunisian families (FT13 and FT20, Ben Said et al., 2016) and the Moroccan NSHL7 family (in orange, this study) on chromosome 5q23.2-q32 is reported, and the shared region containing *SLC22A4* is shown. The image is adapted from the UCSC Genome Browser.

Parametric multi-point data analysis evidenced a unique strong linkage signal peak (LOD score = 3.716, Supplementary Figure S1) shared by the affected siblings and spanning an interval of about 3.2 Mb on chromosome 5q23.3–q31.1 (chr5:128789554–131970885, hg19 Human reference genome coordinates), between markers rs11241999 and rs2237060 (Figures 1A, 2).

### Exome Sequencing Identifies the rs768484124 (SLC22A4 c.338G>A) Variant Within the Critical Linkage Region

To confirm linkage results and identify the specific genetic determinant of the disease in the family, we sequenced the exome of the IV2 and IV4 affected siblings. Approximately 5 Gb of high-quality sequence data mapping to the exome-targeted regions were generated as 75 bp paired-end reads. Nearly 82% of the intended target was covered with an average depth of 30× (Table 1). Exome data were analyzed to (1) verify the coverage and presence of candidate pathogenic variants in known ARSNHL genes, with particular reference to known deafness-associated North African mutations (Chakchouk et al., 2015); (2) detect potentially pathogenic variants within the linkage region on chromosome 5; and (3) explore the presence of likely pathogenic variants (either in the homozygous or compound heterozygote state) also outside the linkage region.

**TABLE 1:** Exome sequencing statistics.

	IV2	IV4	Mean
Total number of bases (Gb)	5.22	5.54	5.38
Total number of HQ bases (Gb)	4.80	5.09	4.95
Number of reads (M)	62.86	66.85	64.86
Reads mapped to target ± 100 bp	53,500,171	57,078,787	55,289,479
Target size (Mb)	47.23	47.23	47.23
Mean coverage	76.55	81.77	79.16
Covered 1× (%)	98.68	98.69	98.68
Covered 10× (%)	94.37	94.57	94.47
Covered 20× (%)	88.42	89.13	88.78
Covered 30× (%)	80.95	82.33	81.64

**M, million; HQ, high quality.**

None of the 58 pathogenic variants included in the North African Deafness (NADf) chip (Chakchouk et al., 2015) was present in our patients. In addition, no candidate pathogenic variant was detected in 88 known autosomal-recessive or autosomal-dominant SNHL genes, after verifying coverage of the entire coding sequence in exome data. Sequencing data were not available/reliable for four NSHL genes (*STRC*, *OTOA*, *OTOG*, and *COL11A2*), at least two of which are well known to have mapping issues due to the presence of segmental duplications and/or pseudogenes (Supplementary Table S1; Mandelker et al., 2014). Within the critical linkage region on chromosome 5, exome data analysis identified 48 and 52 homozygous variants in individual IV2 and IV4, respectively. Of these, 45 were shared between the affected siblings: 9 were located within gene exons and 6 were non-synonymous (Supplementary Table S2). Among non-synonymous variants, only two were rare in the general population (minor allele frequency ≤ 1%) and had a CADD score ≥ 20: rs768484124 within *SLC22A4* (NM_003059.2:c.338G>A; NP_003050.2:p.C113Y) and rs192638050 within *P4HA2* (NM_004199.3:c.1316G>A; NP_004190.1:p.R439Q). *SLC22A4* was the most likely candidate NSHL gene, as the NM_003059.2:c.338G>A (p.C113Y) substitution within exon 1 was previously reported as a deafness-causing mutation (Ben Said et al., 2016). The variant has an estimated allele frequency of 2.185 × 10^–5^ in the Genome Aggregation Database (gnomAD v.2.1.1, last accessed July 2020)^3^, being present—always in the heterozygous state—in 5 out of 114,412 individuals. The highest frequency (1.212 × 10^–4^) is reported in the Latino/Admixed American population (4 heterozygotes over 33,010 analyzed subjects). The *P4HA2* p.R439Q variant has also been reported in the deaf family described by Ben Said et al. (2016) but was excluded as the cause of hearing loss because it is classified as benign or tolerated by several predictors (such as SIFT and Polyphen-2) and affects a residue that is not conserved in the mouse *P4ha2* ortholog (NP_035161). Indeed, the mouse protein contains a glutamine (p.Gln441), which corresponds to the amino acid substitution found in humans.

The *SLC22A4* c.338G>A (p.C113Y) variant was found in the homozygous state in all six affected NSHL7 family members, as confirmed by Sanger sequencing; unexpectedly, the variation was identified, again in the homozygous state, also in the IV1 normal-hearing sibling, suggesting incomplete penetrance of the hearing defect. The normal-hearing brother indeed shares the same haplotype on chromosome 5 linkage region with his affected siblings, as well as the variant genotype at the *P4HA2* c.1316 position. In addition, the *P4HA2* p.R439Q is present in the homozygous state in the normal-hearing IV3 subject. Conversely, the *SLC22A4* c.338G>A substitution (rs768484124) is absent in a cohort of 125 Italian audiologically tested normal-hearing controls (mean age at withdrawal 32 ± 9 years) and in the ∼1,000 individuals from the GME Variome study cohort (http://igm.ucsd.edu/gme/data-browser.php, last accessed July 2020), which includes subjects from the North African population (85 from the Northwest African region, 423 from Northeast Africa) (Scott et al., 2016). As comparison, the *P4HA2* p.R439Q variant (rs192638050) is present in the heterozygous state in two subjects from Northeast Africa. Finally, screening of the entire *SLC22A4* gene (10 coding exons and adjacent splice junctions) in seven additional unrelated ARSNHL patients of North African origin (two from Morocco, four from Egypt, and one from Tunisia) did not detect any putative pathogenic variant.

### No Additional Candidate Pathogenic or Modifier Variants Are Detected by In-Depth Exome Analysis

To investigate deeper the reason why not all subjects carrying the *SLC22A4* c.338G>A (p.C113Y) variant manifest the hearing phenotype, we could hypothesize that affected siblings carry additional variants (outside the critical linkage region) with additive/synergic pathogenic effect. If this would be the case, we might expect that all NSHL-affected individuals would carry such modifier variant(s). However, we were not able to detect a second linkage region segregating with the phenotype in our family. In any case, we re-analyzed all potential pathogenic variants detected by exome sequencing independently on the affected siblings and then focused on those shared between the two of them. Only non-synonymous/splicing variants with MAF ≤ 1% in GnomAD/Exac were selected, and an autosomal recessive mode of inheritance was hypothesized. In addition, we excluded genes located within ENCODE Blacklist regions (e.g., *MUC4*, *MUC6*, *ZNF806*, *ANKRD36*, and *ANKRD36B*), as they likely represent next-generation sequencing artifacts, with high signal independent from cell line, experiment, or the specific phenotype under study (Amemiya et al., 2019). A total of 48 variants in 25 genes were found, including *SLC22A4* c.338G>A (p.C113Y) and *P4HA2* c.1316G>A; (p.Arg439Gln) (Supplementary Table S3). Among these, 14 variants in 12 genes (10 genes carrying a candidate homozygous variant and 2 genes carrying two different heterozygous variants) had a predicted CADD score (v1.3) ≥ 20 and a frequency in GME population ≤ 1% (Supplementary Table S4). Notably, 7 of the 12 shortlisted genes are located on chromosome 5, although only 2 (*SLC22A4* and *P4HA2*) fall within the linkage peak. Of these, in addition to the variants in *SLC22A4* and *P4HA2*, we evaluated by Sanger sequencing in all available family members the segregation with hearing loss of the variants in *SNX2*, *IK*, *PCDHB3*, and *PCDHB16*. The *SNX2* NM_003100:c.934C>A (p.Q312K) does not segregate with NSHL in the right branch of the pedigree, being the affected IV7 subject wild type. The *IK* NM_006083:c.965T>C (p.I322T), *PCDHB3* NM_018937:c.242C>T (p.T81L), and *PCDHB16* NM_020957:c.1024G>C (p.D342H) variants are present in the homozygous state not only in all affected family members but also in two normal-hearing subjects (IV1 and IV3). Hence, further exploration of exome data coupled with segregation analyses again confirmed as the most likely pathogenic variant the *SLC22A4* c.338G>A (p.C113Y), rs768484124.

## Discussion

Hearing loss is the most common sensory disorder in the world, affecting about 466 million individuals (data from the World Health Organization website: https://www.who.int/news-room/fact-sheets/detail/deafness-and-hearing-loss, last accessed September 2020). The majority of cases are due to genetic factors, with more than 1,000 mutations identified in about 100 different genes (Rudman et al., 2017). In addition to this extreme genetic and allelic heterogeneity, the frequency of the mutations associated with deafness can vary markedly according to the specific ethnic group considered, further complicating the genetic diagnosis. In general, the mutational landscape of NSHL in North Africans is only partially overlapped with the one found in Caucasians; in particular, *GJB2/GJB6* mutations are relatively uncommon. Indeed, SNHL-causing mutations identified in North African countries currently involve only a small set of genes, i.e., *GJB2, MYO7A, MYO15A, SLC26A4, TMC1, TMPRSS3, DFNB31, ESRRB, ESPN, DFNB59, LRTOMT, LHFPL5, PNPT1, TPRN*, and *MT-RNR1* (Chakchouk et al., 2015). Besides the bias due to the low number of genetically analyzed patients, this could also be related to the frequency of consanguineous marriages among North African populations, which inevitably leads to a clustering of rare recessive genetic defects (Rudman et al., 2017).

In 2016, a novel gene, i.e., *SLC22A4*, was identified as responsible for ARSNHL in two unrelated consanguineous families of Tunisian ancestry. The two families shared the same pathogenic variant, suggesting a founder effect, as confirmed by haplotype analysis (Ben Said et al., 2016). In the present study, we report a third ARSNHL family of Moroccan origin, carrying the same NM_003059.2:c.338G>A (p.C113Y) missense variant (rs768484124), thus providing the first independent replication of the involvement of *SLC22A4* in hearing loss. Interestingly, Northwest African samples from the GME study (comprising individuals from Morocco, Algeria, and Tunisia) were among the least admixed ones, compared with other subregions, suggesting that these three countries represent a unique founder population (Scott et al., 2016). Considering that all NSHL7 individuals carrying the *SLC22A4* c.338G>A substitution also carry the *P4HA2* c.1316G>A variant previously reported in the Tunisian families, and they share the same allele for the D5S2115 microsatellite marker (Figure 1A), we can hypothesize that the p.C113Y substitution is on a founder haplotype with a broader distribution than previously thought, and its screening might be valuable at least in patients/families with known Northwest African origin.

Compared to the first family (FT13) described (Ben Said et al., 2016), the deaf individuals from NSHL7 family show a similar hearing defect (bilateral, symmetric, all tones affected), but a later onset, as they had an adequate speech development. In addition, the IV4 subject displays a less severe impairment compared to both his older brother and the FT13 family members (Table 2). It should be noted that the p.C113Y variant in *SLC22A4* was also found, in the homozygous state, in a normal-hearing NSHL7 family member, which suggests incomplete penetrance. Indeed, incomplete/reduced penetrance of the hearing phenotype in humans has been reported (Vona et al., 2015), but few molecular studies have been performed to explain this phenomenon. Attempts were made to identify modifier genes; an example is represented by *DNFM1*/*METTL13* (Riazuddin et al., 2000; Yousaf et al., 2018). By analogy with *DNFM1*/*METTL13*, which acts as a dominant suppressor of recessive *DFNB26*/*GAB1* deafness, it is intriguing to speculate about the existence of a dominant modifier of *SLC22A4*, which could suppress the onset of deafness in the non-penetrant individual from family NSHL7. Of course, the genetic mapping of such a modifier is currently unfeasible due to the limited number of subjects with non-penetrant phenotype associated with the *SLC22A4* p.C113Y variant. More in general, further studies in wider patient populations with different ethnic backgrounds are needed to assess the impact of mutations in *SLC22A4* gene in NSHL.

**TABLE 2:** Clinical features of patients carrying the SLC22A4 p.Cys113Tyr mutation.

	FT13 [4]	FT20 [4]	NSHL7 (This study)
Origin	Tunisia	Tunisia	Morocco
Age of onset	Prelingual	n.a.	Post-lingual (normal speech development)
Hearing status	Bilateral symmetric impairment, all tones affected	Bilateral symmetric impairment, all tones affected	Bilateral symmetric impairment, all tones affected
Severity	Profound	n.a.	Moderate to profound
Progression	No	n.a.	Yes
Penetrance	100% (6/6)	n.a.	85% (6/7)

Little is known about the role of *SLC22A4* in normal hearing and deafness. *SLC22A4* codes for OCTN1, an organic cation transporter that is ubiquitously expressed in the body (Yabuuchi et al., 1999). In the inner ear, it was demonstrated to have a diffuse expression across the epithelia, including the hair cells and the apical surface plasma membrane of the endothelial cells of the stria vascularis.

A first insight into *SLC22A4* function derives from the experiments demonstrating that the p.C113Y missense variant alters the ability of the OCTN1 transporter to uptake the antioxidant ERGO, and consequently affects the fatty-acid-based metabolic energy production pathway. Starting from this finding and considering the protein’s pattern of expression, the authors speculate that OCTN1 could be indispensable for carnitine and ERGO transport into the inner ear (Ben Said et al., 2016).

This hypothesis is further supported by the data from knockout animals: in *Caenorhabditis elegans*, knockout for OCT-1, the orthologous of mammalian OCTN1, led to a significant elevation of oxidative damage, with a consequent shortening of lifespan (Cheah et al., 2013), whereas a metabolome analysis of blood and several organs of *octn1*^–/^*^–^* mice indicated complete deficiency of ERGO and greater susceptibility to intestinal inflammation (Kato et al., 2010). More recently, the phenotypic description of a *SLC22A4* knockout mouse (*Slc22a4*^*tm1.1(KOMP)Vlcg*^ allele)^4^ was made available by the International Mouse Phenotyping Consortium:^5^ the evoked auditory brain stem responses are not significantly different between wild-type mice and homozygous mutants, indicating no apparent signs of hearing loss. Although the number of analyzed animals was very limited, and the panel of experiments to test the hearing function could be certainly broadened, these results do not help in shedding light on the role of the *SLC22A4*/OCTN1 transporter in the normal hearing function. Again, it would be interesting to evaluate the impact of different genetic backgrounds on the expressivity of *SLC22A4*-dependent hearing phenotype. However, given the lack of phenotype in the *Slc22a4*^*tm1.1(KOMP)Vlcg*^ and the presence of the variant at the homozygous state in one normal-hearing individual, we cannot completely exclude the following: (1) *SLC22A4* is the causative gene but the rs768484124 is not the deafness-associated variant or (2) *SLC22A4* is not the causative gene and does not contribute to hearing function. In the first hypothesis, the SNHL-causing variant(s) could regulate *SLC22A4* function, whereas in the second, the variant(s) could be in linkage disequilibrium with rs768484124. In both cases, such variants could have escaped our analysis for some reasons (e.g., regulatory variants in untranslated regions, promoter, or non-protein coding genes; deep-intronic splicing variants missed by exome sequencing).

## Conclusion

In conclusion, the present study confirms the involvement of *SLC22A4* in ARSNHL, and highlights the recurrence of the p.C113Y mutation at least in the Northwest African population, where it likely represents a founder NSHL mutation. In addition, we report the possibility of an incomplete penetrance of the hearing phenotype associated with the *DFNB60* locus.

## Data Availability Statement

The datasets presented in this article are not readily available for ethical and privacy reasons related to the publication of identifiable genetic information. Requests to access the datasets should be directed to the corresponding author.

## Ethics Statement

The studies involving human participants were reviewed and approved by Ethical Committees of the Niguarda Ca’ Granda Hospital, Milan, Italy and Fondazione IRCCS Cà Granda Ospedale Maggiore Policlinico, Milan, Italy. Written informed consent to participate in this study was provided by the participants themselves or by their legal guardian/next of kin.

## Author Contributions

GS, CC, RA, and SD conceived and designed the experiments. MR performed array-based genotyping and linkage analyses. CC and GS conducted exome sequencing analyses. CC, LM, and PP performed the mutational screening by Sanger sequencing. CC designed and performed HRM assay. UA, PP, PC, and LM were responsible for patient clinical and family history evaluation, collection of blood samples, DNA extraction, and patients’ follow-up. GS, CC, and MR drafted the manuscript, tables, and figures. GS supervised the entire study. All authors reviewed the manuscript. All authors contributed to the article and approved the submitted version.

## Conflict of Interest

The authors declare that the research was conducted in the absence of any commercial or financial relationships that could be construed as a potential conflict of interest.

## Footnotes

1 http://hereditaryhearingloss.org/

2 https://www.uniklinikum-leipzig.de/einrichtungen/medizinische-klinik-3/Seiten/easylinkage-download.aspx

3 http://gnomad.broadinstitute.org/

4 http://www.mousephenotype.org/data/genes/MGI:1353479

5 http://www.mousephenotype.org/

## Supplementary Material

Supplementary Figure 1 Linkage maps obtained by parametric multi-point data analysis with GeneHunter v2.1r5. The *y*-axis indicates the LOD score value and the *x*-axis the position on the chromosome(s). **(A)** Linkage map displaying the results of the multi-point analysis performed on all chromosomes. Computation in sets of 100 markers, spacing 1 cM. **(B)** Linkage map of the multi-point analysis focused on chromosome 5. Computation in sets of 100 markers, spacing 0.1 cM.

Supplementary Table 1 Coding sequence coverage of 92 known NSHL-causing genes in exome data.

Supplementary Table 2 Exome variants within the critical linkage region and shared between the two affected siblings.

Supplementary Table 3 List of exome-derived candidate non-synonymous/splicing variants shared among the two analyzed siblings. SIFT: T, tolerated; D, deleterious; Polyphen: B, benign; PD, probably damaging. FLAG, FrequentLy mutAted Gene, as described by Shyr et al. (2014, 2017).

Supplementary Table 4 Shortlist of top 12 exome-derived candidate genes and variants shared among the two analyzed siblings. SIFT: T, tolerated; D, deleterious; Polyphen: B, benign; PD, probably damaging. FLAG, FrequentLy mutAted Gene, as described by Shyr et al. (2014, 2017).
