# Supplementary material for: SLC22A4 Gene in Hereditary Non-syndromic Hearing Loss: Recurrence and Incomplete Penetrance of the p.C113Y Mutation in Northwest Africa
Source: Front Genet. 2021 Feb 10;12:606630. doi: 10.3389/fgene.2021.606630 (PMC7902881; doi:10.3389/fgene.2021.606630)
Supplement: Supplementary Figure 1 — Linkage maps obtained by parametric multi-point data analysis with GeneHunter v2.1r5. The y-axis indicates the LOD score value and the x-axis the position on the chromosome(s). (A) Linkage map displaying the results of the multi-point analysis performed on all chromosomes. Computation in sets of 100 markers, spacing 1 cM. (B) Linkage map of the multi-point analysis focused on chromosome 5. Computation in sets of 100 markers, spacing 0.1 cM. [file Image_1.PDF]

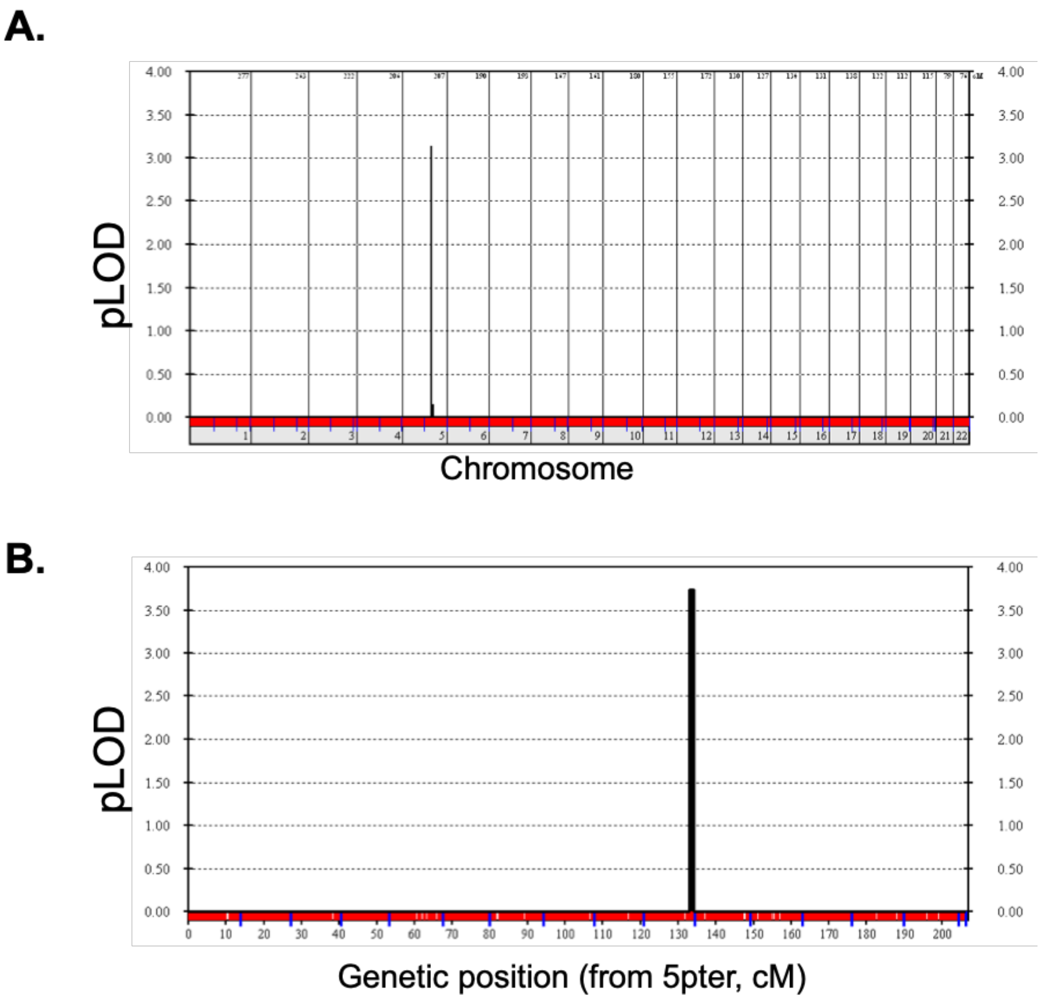

Supplementary Figure 1. Linkage maps obtained by parametric multi-point data analysis with GeneHunter v2.1r5. The y-axis indicates the LOD score value and the x-axis the position on the chromosome(s). **A.** Linkage map displaying the results of the multi-point analysis performed on all chromosomes. Computation in sets of 100 markers, spacing 1 cM. **B.** Linkage map of the multi-point analysis focused on chromosome 5. Computation in sets of 100 markers, spacing 0.1 cM.
